# Random Forest Modelling of High-Dimensional Mixed-Type Data for Breast Cancer Classification

**DOI:** 10.3390/cancers13050991

**Published:** 2021-02-27

**Authors:** Jelmar Quist, Lawson Taylor, Johan Staaf, Anita Grigoriadis

**Affiliations:** 1 Cancer Bioinformatics, Cancer Centre at Guy’s Hospital, King’s College London, London SE1 9RT, UK; jelmar.quist@kcl.ac.uk (J.Q.); lawsontaylor@hotmail.co.uk (L.T.); 2 School of Cancer and Pharmaceutical Sciences, King’s College London, London SE1 1UL, UK; 3 Breast Cancer Now Research Unit, Cancer Centre at Guy’s Hospital, King’s College London, London SE1 9RT, UK; 4 Division of Oncology, Department of Clinical Sciences Lund, Lund University, Medicon Village, SE-223 81 Lund, Sweden; johan.staaf@med.lu.se

**Keywords:** breast cancer, random forest, machine learning, integrative analysis, DNA damage repair

## Abstract

**Simple Summary:**

Breast cancer is a complex disease, and the identification of its underlying molecular mechanisms is critical for the development of treatment strategies. The purpose of this study was to implement a computational framework that is capable of combining many types of data into a meaningful classification. While our approach can be used on many types of data and in many diseases, we applied this framework to breast cancer data and identified six triple-negative breast cancer subtypes with distinct underlying molecular mechanisms. The relevance of our approach is highlighted by the clinical outcome analysis in which a group of patients responding poorly to standard-of-care adjuvant chemotherapy was identified. This study serves as a starting point for our computational framework, which can be extended to different types of data from different diseases.

**Abstract:**

Advances in high-throughput technologies encourage the generation of large amounts of multiomics data to investigate complex diseases, including breast cancer. Given that the aetiologies of such diseases extend beyond a single biological entity, and that essential biological information can be carried by all data regardless of data type, integrative analyses are needed to identify clinically relevant patterns. To facilitate such analyses, we present a permutation-based framework for random forest methods which simultaneously allows the unbiased integration of mixed-type data and assessment of relative feature importance. Through simulation studies and machine learning datasets, the performance of the approach was evaluated. The results showed minimal multicollinearity and limited overfitting. To further assess the performance, the permutation-based framework was applied to high-dimensional mixed-type data from two independent breast cancer cohorts. Reproducibility and robustness of our approach was demonstrated by the concordance in relative feature importance between the cohorts, along with consistencies in clustering profiles. One of the identified clusters was shown to be prognostic for clinical outcome after standard-of-care adjuvant chemotherapy and outperformed current intrinsic molecular breast cancer classifications.

## 1. Introduction

Breast cancer, like many other complex diseases, is molecularly heterogeneous and advancements in breast cancer classification have been key to improve diagnosis, treatment and prognosis. Large amounts of data, including genomic (e.g., single nucleotide variations, structural and numerical copy number alterations), transcriptomic (expression of single genes and gene signatures), and epigenetic features (e.g., methylation), are being generated as part of sophisticated studies [1,2,3]. From a clinical perspective, breast cancers are classified based on the expression of oestrogen receptor (ER), progesterone receptor (PgR), and human epidermal growth factor receptor 2 (HER2). Few consortia have uncovered key molecular features in breast cancers, including amplification of *HER2*, *CCDN1* and *MYC*, and mutations in *TP53*, *PIK3CA* and *PTEN* [1,2,3]. Historically, molecular classifications are often focused on a single biological entity. To begin with, gene expression profiling has led to an internationally accepted intrinsic molecular breast cancer classification system of five subtypes based on a hierarchical clustering method [4,5,6]. This classification was standardised using 50 genes and is now commonly referred to as PAM50 [4]. Using the NanoString nCounter platform (NanoString Technologies, Seattle, WA, USA), an FDA approved assay was developed to calculate a risk-of-recurrence score able to identify late (>5 years) recurrences in postmenopausal women with ER-positive breast cancer receiving adjuvant therapy [7].

Several breast cancer classifications have since followed. In 2016, Nik-Zainal and colleagues [3] demonstrated that unsupervised hierarchical clustering on rearrangement signatures in cancer genomes, each representing distinct mutational processes, can segregate breast cancers into seven clusters. Amongst those was a group of breast cancers with a high proportion of rearrangement signature 3, characterised by small tandem duplications (<10 kb), and immunohistochemically defined as triple-negative breast cancer (TNBC); i.e., lacking expression of ER, PgR, and HER2. The inactivation of *BRCA1* through genetic or epigenetic alterations occurs in approximately 35% of all TNBCs [8], leads to defective homologous recombination (HR), an error-free DNA damage repair mechanism, and results in increased levels of distinct types of genomic instability, including a small tandem duplicator phenotype [9,10,11,12,13,14,15]. Only a few studies have focused on classification approaches tailored specifically for TNBC. Lehmann and colleagues [16,17] identified four TNBC subtypes based on gene expression profiling, whereby the basal-like 1 subtype showed the highest pathological response rates to neoadjuvant chemotherapy [17]. A more recent study constructed a four-gene decision tree signature [18]. Of the six subtypes, MC6-TNBCs were found to have an improved response to neoadjuvant platinum-based chemotherapy.

With the increasing availability of omics data, multimodal models have gained more traction. For instance, ten breast cancer subgroups with distinct clinical outcomes were described based on a joint latent variable model on gene expression and copy number alterations [2,19]. We have previously derived six TNBC subgroups by employing a Bayesian methodology integrating gene expression and copy number alterations; one of the subgroups predicted sensitivity to platinum-based chemotherapy in TNBC patients with metastatic disease [18]. Since HR deficient breast cancers, including TNBCs, respond to therapeutic strategies exploiting DNA repair deficiency (e.g., platinum salts and poly(ADP-ribose) polymerase (PARP) inhibitors [20]), classification approaches can carry important clinical consequences for the improvement of patient stratification and treatment strategies.

To unravel complex patterns from biological data, machine learning methods can be used. The two primary types of machine learning are unsupervised and supervised. Unsupervised machine learning is frequently used for subgroup discovery where labels for data are unknown. In contrast, with supervised machine learning, one seeks to determine the relationship between a set of input features and their corresponding labels. Beyond the accuracy of this relationship, feature selection is an important process in supervised machine learning and provides insight into the underlying biology of the data labels which is crucial for the translational interpretation.

Random forest (RF), an example of supervised machine learning, has become one of the most widely used methods by offering a broad range of solutions to classification problems, and is known for its high predictive accuracy and ability to handle high-dimensional and mixed-type data [21]. The concept of feature selection and variable importance measures (VIMs) is an implicit technique performed in RF and is assessed by the Gini impurity criterion index [22]. This index provides a measure of the prediction power of features in classification problems based on the principle of impurity reduction [23]. VIMs play an essential part in the identification of biologically relevant biomarkers [24,25,26,27], but can become unreliable in high-dimensional and mixed-type data. In cancer classification, this is often the case when gene expression (real-valued continuous), copy number alterations (integer) and clinical characteristics (categorical/binary) are integrated. As such, features of high cardinality are more likely to be favoured and binary features that contain information that could predict a data label are ignored [21].

To address this inherent bias in RF, an alternative approach was introduced known as conditional inference forest (CIF). Instead of relying on the Gini impurity criterion index as a splitting criterion [28], this approach performs permutation tests on the features and data labels [29]. As a result, features of high cardinality are no longer favoured and reliable VIMs can be retrieved. However, even with the CIF framework, the variable importance is only unbiased under a nonreplacement subsampling scheme implemented during the forest construction [28]. Similarly, building on RF, a regularised tree framework was proposed that introduces a penalty when the information from a new splitting criterion is similar to that of a feature that has already been split on previously. The idea of regularised random forest (RRF) is to limit the size of the tree and provide a simpler and ideally a more generalisable model that is less prone to overfitting [30].

Our objective was to implement a permutation-based framework for RF, RRF and CIF, with the aim of classifying complex high-dimensional mixed-type data. Initial simulation and benchmarking studies were performed to demonstrate the robust performance of the permutation-based framework and compare the different RF approaches. Proof of concept application to the rearrangement signatures of the breast cancer data revealed that the identified subgroups were compact and homogenous, meaning the majority of samples belong to one feature category. This is particularly important in cancer classification as it aids in the translational interpretation and applicability. We evaluated the permutation-based framework on mixed-type data from two breast cancer cohorts and observed that particularly the CIF approach results in distinct subgroups. Finally, we provide an extensive qualitative and quantitative evaluation of the identified subgroups and interpret these in the context of a translational study.

## 2. Results

### 2.1. Implementation of Permutation-Based Random Forest Classification

Our permutation-based approach for the classification of mixed-type data builds on an algorithm for deriving a forest distance [31]. Briefly, a synthetic dataset, composed of half synthetic and half real data, is generated by independently sampling from the real data. To derive a between-sample similarity measure, RF, RRF or CIF is applied. A proximity matrix is then calculated and subsequently converted into a dissimilarity matrix. Traditional clustering algorithms are used to group data. VIMs are calculated and reported for each feature and can be used to investigate the translational implications of the classification. A diagram illustrating the permutation-based random forest classification, as implemented in the statistical environment R [32], is provided in Figure S1.

#### 2.1.1. Cardinality in Simulation Studies

**Figure 1:**
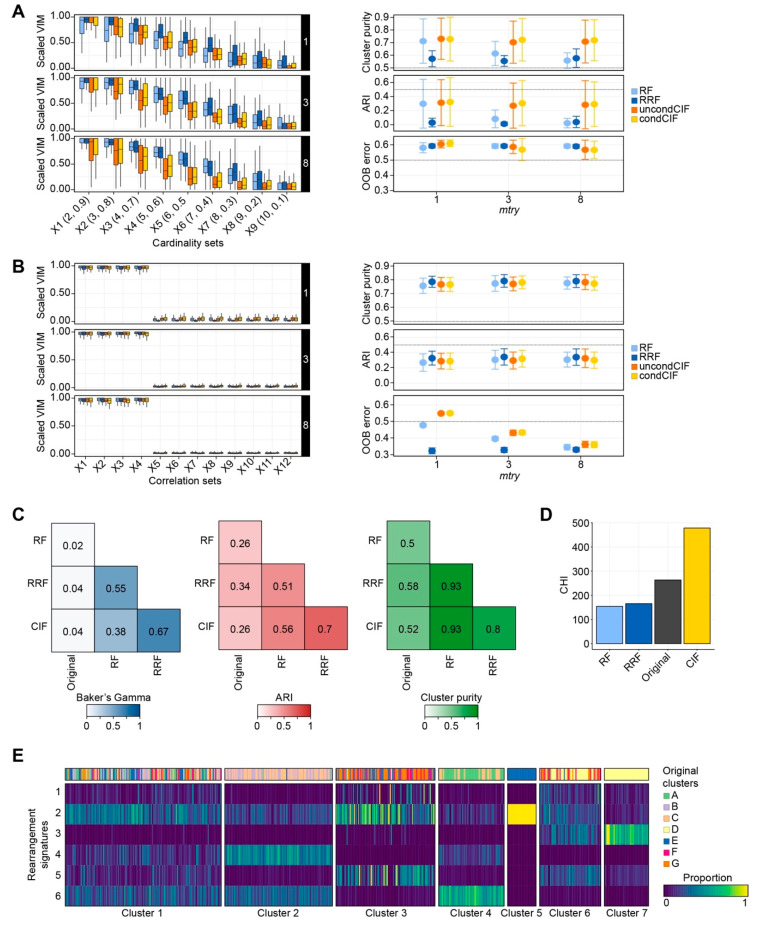
Performance of permutation-based random forest classification. (**A**) (left) Boxplot depicting scaled variable importance measures (VIMs) (y-axis) for random forest (RF) (light blue), regularised random forest (RRF) (dark blue), unconditional conditional inference forest (uncondCIF) (orange) and conditional CIF (condCIF) (yellow) in different cardinality simulation studies (x-axis). Performance of each permutation-based RF method was assessed by varying the number of features available for a split point (*mtry* of 1, 3 and 8). (right) Cluster purity, ARI (Adjusted Rand Index) and out-of-box (OOB) error for the various RF methods. The error bars depict the distribution of each measure. (**B**) As Figure A, but for the correlation simulation studies. (**C**) The permutation-based random forest classifications (RF, RRF and CIF) were applied to six rearrangement signatures across 560 breast cancers and compared with the original clusters [3]. Level plots for Baker’s Gamma (blue), ARI (red) and Cluster purity (green) were used to compare the different classifications. (**D**) Barplot of the CHI measures (y-axis), representing the overall quality, defined as tightly packed and well-separated clusters given numerical data input, for each of the classifications (x-axis). (**E**) Heatmap of rearrangement signatures using permutation-based CIF. Rows are ordered by the tree derived from the consensus clustering on the CIF obtained forest distance. Seven clusters were found, each annotated with respect to the original clustering [3].

A large number of features in translational research are recorded as categorical data, such as clinical stage and histological subtype. To assess the performance of the permutation-based approach in regard to categorical data, we evaluated cardinality estimates in simulation studies (Figure 1A). For RF and RRF, increasing the number of features available for splitting at each tree node (referred to as *mtry*), had an adverse effect on the model’s ability to identify the relevant predictive features. This resulted in high cardinality, hampered accurate VIM estimation, and therefore negatively influenced the final clustering. For both uncondCIF and condCIF, the latter containing a nonreplacement subsampling scheme to measure variable importance, the relevant predictors increased with a larger *mtry* value, although with expanding variance. While the out-of-box (OOB) error was high across all forests in this simulation design, both CIF methods performed best in retrieving the true labels, as indicated by the Adjusted Rand Index (ARI), and derived clusters with 70% cluster purity.

#### 2.1.2. Correlation Bias in Simulation Studies

Correlated features are a common problem in high-dimensional data and can negatively impact feature selection. Using simulation studies, the ability of the permutation-based approach in handling such features was assessed. VIMs for all forests did not reflect the coefficient values simulated by the data generating process (Figure 1B). Specifically, the VIMs for the correlated features dominated, contradicting the classification setting previously proposed [29,33]. Note that correlation set $X_{4}$ ($\beta_{4}=0)$ is given equal importance as its correlated features $X_{1}$,…,$\;X_{3}$, while features $X_{5}$,…,$\;X_{7}\;$are given near-zero importance despite their coefficients ($\beta_{5}=-5$, $\beta_{6}=-5$ and $\beta_{7}=-2$, respectively). This indicates that the properties of a permutation scheme do not translate directly from supervised to unsupervised machine learning and may impede the interpretation of variable importance in the correlated setting. To note, correlated features could represent biological relationships that may be of clinical importance and as such, preference may be given to a standard permutation scheme. With respect to the clustering metrics, all four permutation-based approaches performed equally, were independent of the number of features available for splitting at each tree node (*mtry*), and generated highly pure clusters with mean ARI measures of 0.3; the latter suggesting that the clustering produced is nonoptimal in absolute terms but remained constant (Figure 1B).

#### 2.1.3. Performance Evaluation Using Real-World Datasets

The prior simulation studies were designed to query the performance of the permutation-based framework in a pure feature space. We, therefore, further evaluated our approach using data from the UCI Machine Learning Repository [34]. A total of nine datasets were obtained, either with a pure feature space (e.g., only-categorical or only-numerical) or mixed-type data. The ARI for only-numeric and only-categorical data was by far superior to any mixed-type datasets (Table S1). In seven out of the nine datasets, RRF performed best, including all three mixed-type datasets. Cluster purity, the extent of which clusters contained one class, differed less amongst datasets and methods, and only showed a slight superiority for CIF. To assess how well each method grouped samples based on their numeric or categorical features, the Calinski–Harabasz Index (CHI) and feature purity were calculated. The CHI measure indicated a high concordance for CIF classification in all three numeric-only datasets. Similarly, CIF performed best with regards to feature purity and was superior in all but one of the datasets containing categorical data. In the mixed-type datasets, CIF performed best on the Liver dataset, which consists predominantly of numeric features, and slightly better than RRF on the Census dataset. RRF performed best on the Credit dataset and produced a relatively concordant classification of the numeric features.

### 2.2. Classification of Breast Cancers Based on Rearrangement Signatures

The performance evaluations above demonstrated that the permutation-based framework can be employed for classification purposes. Next, we asked how these methods perform on molecular data obtained from 560 breast cancer patients, referred to as ICGC [3]. By hierarchical clustering on six rearrangement signatures, seven clusters were previously uncovered, some of which were associated with distinct clinical characteristics (e.g., rearrangement signature 3 and *BRCA1* mutations). We tested our permutation-based approach on the six rearrangement signatures and compared the results with the classification reported in the publication. The dendrograms of RRF and CIF were most similar (Baker’s Gamma = 0.67), followed by the dendrograms of RF and RRF (Baker’s Gamma = 0.55). The dendrogram from Nik-Zainal and colleagues [3] and each of our forest-constructed dendrograms showed little similarity (Barker’s Gamma = 0.02–0.04) (Figure 1C). The ARI measure indicated that the RRF implementation produced labels most similar (ARI = 0.34) to the original clustering. With an ARI of 0.7, the clustering labels produced by RRF and CIF were the most similar. The predicted labels from RF, RRF and CIF were similar to the original cluster labels (cluster purity of 0.50, 0.58 and 0.52, respectively). Baker’s Gamma was highest between RRF and CIF, while cluster purity showed high concordance between RF and RRF, as well as RF and CIF.

Overall, the permutation-based forest clustering algorithms produced dendrograms and cluster labels distinct from the original clustering labels. In concordance to the published clustering, both RRF and CIF derived seven clusters, whilst RF identified only five clusters. In terms of feature selection, RF and RRF were in agreement with ranking rearrangement signature 6 as the most informative feature (Table S2). In contrast, CIF selected rearrangement signature 3 as the most informative feature, a signature particularly observed in *BRCA1*-deficient cancers [3,9]. For each approach, the CHI score was calculated to measure how tightly packed these clusters were given the numerical data. The original clustering achieved a CHI score of 263. Both RF and RRF performed relatively poor (CHI = 154 and 165 for RF and RRF, respectively) (Figure 1D), whilst the permutation-based CIF approach had a CHI score of 478, and thus produced the tightest clusters. By comparing the sample composition of the original with the newly derived CIF clusters (Figure 1E), the highest similarity was observed amongst CIF Cluster #5 and #7 which contained tumours exclusively attributed to the original Cluster E and Cluster D, respectively. CIF Cluster #4 was predominantly comprised of original Cluster A (69.35%), with some assigned Cluster C (30.65%). CIF Cluster #2 was a mix of original Cluster C (50.00%) and Cluster B (47.06%). The main differences were found in CIF Cluster #1, #3, and #6 as their tumour sample composition was a mixture of the original clusters and explained the differences in CHI scores. Thus, CIF produced the most robust clustering using these six rearrangement signatures.

#### 2.2.1. Classification of Breast Cancers by Mixed-Type High-Dimensional Data

Next, we examined how the implemented clustering approaches perform when including 147 mixed-type features (Table S3), including driver gene alterations (binary), clinico-pathological characteristics such as grade (categorical) and age (continuous), and varying measures of genomic instability available for the ICGC data. All features are commonly reported clinical and molecular characteristics that provide information pertaining to cancer biology, treatment and prognosis. While RF identified five clusters (Table S4), RRF (Table S5) and CIF (Table S6) split the data into seven groups. Similar to their performance in real-world datasets, the CIF method produced clusters with the highest feature purity. By inspecting the sample composition within each cluster, the CIF clustering was found to produce clusters highly concordant with IHC status, gene expression-based subtypes (AIMS) and tumour grade. Note that the clusters reported in this section are different from those reported in the previous section, which were established using only rearrangement signatures.

**Figure 2:**
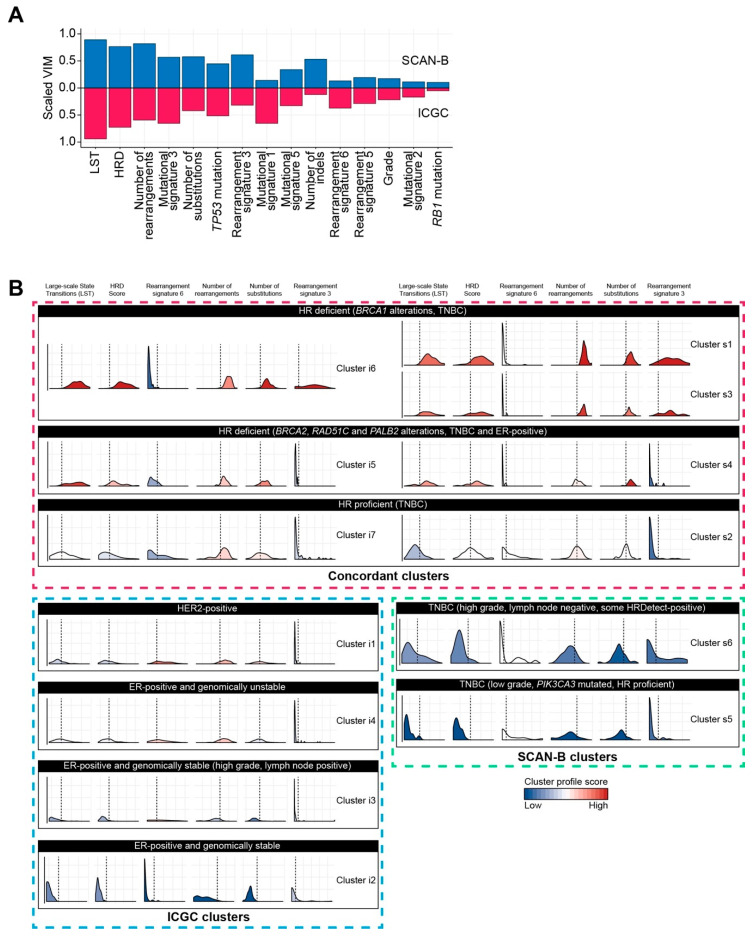
Cluster profile comparison of mixed-type high-dimensional data in ICGC and SCAN-B. (**A**) Barplot of scaled VIMs (y-axis) in SCAN-B (blue) and ICGC (red). In both datasets, condCIF was performed on all available features. The VIMs were scaled and ordered by rank in both datasets. (**B**) Cluster profile scores comparing SCAN-B (cluster s1 to s6) and ICGC (cluster i1 to i7) derived clusters. The distribution of large-scale state transition (LST), homologous recombination deficiency (HRD) score, rearrangement signature 6, number of rearrangements, number of substitutions and rearrangement signature 3 are shown, with the dashed line indicating the median. The colour of the density indicates the median profile score for that cluster, with a red indicating a relative high prevalence of this feature.

To post the challenge of whether the permutation-based forest clustering algorithms could identify robust clusters, we applied our approach to a second breast cancer cohort, named SCAN-B [8]. In contrast to ICGC, only breast cancers of the TNBC subtype were included in SCAN-B. CIF, which in ICGC produced robust clusters, was applied to SCAN-B, comprising of 241 TNBCs and 50 features of various data types (Table S3). As a result, six clusters were produced (Table S6). Although the relative number of features considered informative (VIM > 0) was different between SCAN-B (39 out of 50) and ICGC (68 out of 147), many of the features selected appeared to be similar between the cohorts (Figure 2A). In both cohorts, highly ranked features included the Large-scale State Transition (LST) [11] and the Homologous Recombination Deficiency (HRD) scores [14], both measures of HR deficiency, as well as the measures capturing the level of genomic instability, such as the number of structural rearrangements and substitutions.

**Figure 3:**
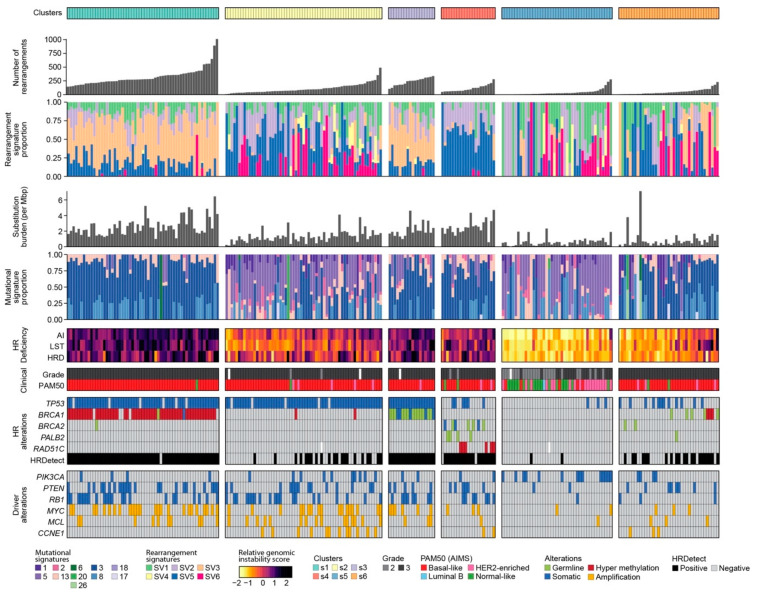
Integrative clusters of triple-negative breast cancer (TNBC). Clusters were derived by applying our permutation-based condCIF to 50 mixed-type features from 241 TNBC patients. Overall levels of genomic instability, as measured by the number of rearrangements and the substitution burden, were different between each cluster. Three clusters (s1, s3, s4) were considered HR deficient, as measured by three genomic signatures (AI, LST and HRD) and HRDetect. Both alterations in HR-related genes, as well as driver genes, varied between the six clusters.

To understand if the clusters represented tumours with similar clinical-pathological and biological characteristics, clusters were manually aligned (Figure 2B). ICGC Cluster i6, and SCAN-B Cluster s1 and Cluster s3 displayed increased frequencies of structural rearrangements and substitutions; features of HR deficiency were prominent, including high levels of LST and HRD, along with rearrangement signature 3. These three subgroups were enriched for HRDetect-positive (HRDetect probability > 0.7) cases (98%, 100% and 99% in Cluster s1 and s3, and i6, respectively) (Figure 3) [8,9]. *BRCA1* silencing, either through mutation or promoter hypermethylation, was observed in 92% of SCAN-B Cluster s1 cases, and 100% of SCAN-B Cluster s3 cases. In ICGC Cluster i6, predominantly consisting of TNBC cases (78 out of 80), *BRCA1* silencing was observed in 30% of the cases. SCAN-B Cluster s4 and ICGC Cluster i5 also exhibited high levels of genomic instability and HR deficiency. In SCAN-B Cluster s4, these features of HR deficiency were the result of a composition of alterations in HR-associated driver genes, including germline (19%) and somatic (14%) mutations in *BRCA2*, promoter hypermethylation of *RAD51C* (29%), and germline *PALB2* mutation (19%). In ICGC Cluster i5, germline *BRCA2* mutations were detected in 86% of the cases; a somatic mutation was observed in an additional two cases and one case had a somatic *PALB2* mutation. Interestingly, 95% of the cases in ICGC Cluster i5 were ER-positive.

In SCAN-B Cluster s2 and ICGC Cluster i7, the latter of which was predominantly triple-negative (94%), tumours exhibited medium levels of genomic instability. In SCAN-B Cluster s2, 34% were considered HRDetect-positive; hypermethylation of *BRCA1* was observed in two cases and 92% of the cases were *TP53* mutated. In ICGC Cluster i7, *TP53* was mutated in 73%. Alterations in HR-associated driver genes were detected in eight cases, with 83% of the cases in ICGC Cluster i7 considered HRDetect-negative. ICGC Cluster i1, generally HER2-positive, had medium levels of genomic instability, whereas Cluster i2 and Cluster i3 were both predominantly ER-positive and genomically stable. ICGC Cluster i4, also ER-positive, was considerably more unstable. In the latter cluster, *TP53* mutations were observed in 53%; in Cluster i3, one sample had a *TP53* mutation. *TP53* was never found mutated in Cluster i2. In both clusters, *PIK3CA* mutations were observed in ~50% of the cases. All four clusters were unique to the ICGC data, which encompassed all breast cancer subtypes. In contrast, Cluster s5 and s6 were unique to SCAN-B. Both clusters were genomically stable. In Cluster s6, few cases (21%) had mutations in HR-associated genes while in Cluster s5 *PIK3CA* mutations were found in 33% versus 10% in Cluster s5 and s6, respectively.

#### 2.2.2. Association of Breast Cancer Classification and Clinical Outcome

**Figure 4:**
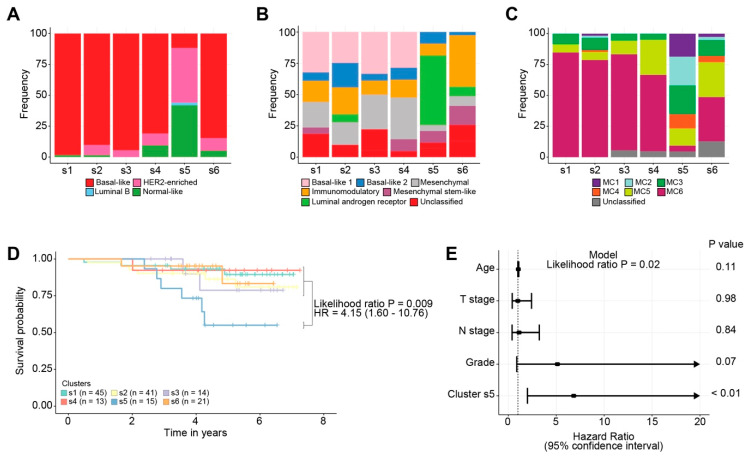
Permutation-based forest clustering derived clusters and clinical outcome in TNBCs from SCAN-B. Barplots illustrate the frequency (y-axis) of (**A**) PAM50, (**B**) TNBCtype, and (**C**) MC subtypes across the different clusters (x-axis). (**D**) Kaplan–Meier analysis of association with overall survival in SCAN-B. Only patients having received adjuvant chemotherapy (*n* = 149) were considered for the analysis. (**E**) Forest plot of the multivariate Cox proportional hazards regression model on overall survival. The covariates that are adjusted in the multivariate model included age, T stage, N stage and grade. Arrows indicate confidence intervals that extend beyond the axis.

Next, we positioned the identified SCAN-B clusters with intrinsic breast cancer classifications, including PAM50 [4,5,6], TNBCtype [16,17] and MC subtypes [18]. Except for Cluster s5, all clusters consisted predominantly of tumours of the basal-like subtype (81–98%) (Figure 4A). As per TNBCtype, 56% of the tumours in Cluster s5 were of the luminal androgen receptor subtype (Figure 4B). This was further supported by an explorative differential gene expression analysis, which identified *AR* as one of the highest ranked differentially expressed genes (Log fold change = 3.7, adjusted *p* value = 7.24 × 10^−12^). It is noteworthy that many of the upregulated genes in Cluster s5 were involved in lipid metabolism. This phenotype is substantiated by a recent study identifying three distinct metabolic states in TNBC [35]. MPS1, denoting the lipogenic subtype, was shown to be more sensitive to inhibitors targeting fatty acid synthesis. Except for Cluster s6, which contained largely immunomodulatory tumours (41%), the remaining clusters had a similar TNBCtype composition. The MC subtypes performed similarly to the PAM50 subtypes, in that the classification was dominated by the MC6 subtype in Clusters s1, s2, s3 and s4 (Figure 4C). Cluster s5 was comprised of a balanced mixture (11–23%) of the remaining MC subtypes.

Previously, the MC6 subtype was shown to be indicative of response to DNA damaging chemotherapeutics [18], and HRDetect-positive tumours had an improved clinical outcome when receiving adjuvant chemotherapy [8]. Since the majority of the patients in SCAN-B received standard-of-care adjuvant chemotherapy (FEC±docetaxel), we hypothesised that patients in Cluster s5 would have a relatively poor outcome. Survival analysis in ICGC was not performed due to the limited availability of outcome and treatment data. Indeed, a univariate Cox proportional hazards regression model confirmed that Cluster s5 had a significantly worse outcome than the remaining clusters (HR = 4.15 (1.60–10.76), Likelihood ratio test *p* value = 0.009) (Figure 4D). In a multivariate model, correcting for age, tumour size, number of positive lymph nodes and tumour grade, Cluster s5 remained a significant prognostic indicator (Figure 4E). AIMS (PAM50), TNBCtype and MC subtypes were not prognostic in univariate models.

## 3. Discussion

The increasing amount of data generated as part of multiomics studies to investigate complex diseases is challenging the current methodologies for data analysis. Consequently, an emerging need for novel or improved algorithms is imminent. In this work, we developed a permutation framework, available on GitHub (see Materials and Methods), for RF, RRF and CIF to find structure in complex high-dimensional mixed-data. We demonstrated our approach by dissecting breast cancers into distinct groups with unique cluster profiles independently in two cohorts. Despite a dissimilarity in size and composition, subgroups and cluster profiles remained robust. In particular, the formation of compact and homogenous clusters is crucial, as these could aid patient stratification, biomarker discovery and ultimately drug target identification.

The applicability of RF approaches as a supervised machine learning technique is well established. For example, CIF was implemented as part of DEMETER, a computational framework employed to model 426 genetic dependencies using 66,646 molecular features, including single nucleotide variations and gene expression [36]. As an unsupervised machine learning technique, RF approaches are less common and often limited to survival analyses [37,38]. In this type of unsupervised analyses, the performance of CIF is superior to RF [37] and has an even higher predictive accuracy when compared with Cox proportional hazards regression models [38]. However, these data do not suffer from the same challenges as the breast cancer classification problem proposed here, which include: (i) large number of predictors and small sample size (p <<n); and (ii) multicollinearity and overfitting, which can influence the accuracy, reproducibility, and interpretation of models. Our permutation-based forest clustering approach for the classification of mixed-type data is implemented with the aim of tackling this (unsupervised) breast cancer classification problem while limiting multicollinearity and overfitting (Figure 1 and Table S1).

A major benefit in the choice of forest methods is the implicit transparency around the feature selection procedure [22]. Not all machine learning approaches report feature importance. In neural networks, a network is generally regarded as a black box and extraction of feature importance can be difficult. Yet depending on the scope of the study, reporting feature selection could be considered a key requirement, especially in translational research. Here, when classifying breast cancers using rearrangement signatures, CIF produced clusters with the highest internal consistency (Figure 1D), whereby rearrangement signature 3 was considered the most informative feature. This genomic signature has been consistently implicated as a measure of HR deficiency [3,9], carries valuable translational information [20], and thus supports the use of CIF over other forest methods. To note, rearrangement signature 6, consistently reported as the most informative in all forest methods (Table S2), is characterised by clustered inversions and deletions that could be the result of kataegis, or a kataegis-like phenomenon [3,39].

Moving to the classification of mixed-type high dimensional data, we found that the CIF approach continued to perform best when including all 147 features in the ICGC data. The tumour composition of identified clusters was concordant with features known to be predictive or prognostic in breast cancer, further supporting the choice of CIF. Robustness is an important criterion in translational research; hence we greatly valued the reproducibility in a second cohort by demonstrating concordance in both feature selection (Figure 2A) and cluster profiles (Figure 2B). The poor overlap between our classification and intrinsic breast cancer classifications (Figure 4A–C) is likely due to the inclusion of genomic features. Composite genomic scars result from mutagenic processes occurring throughout the lifetime of a tumour, thereby complicating the dissection of DNA damage response that is ongoing or historical [40]. In contrast, the transcriptomic landscape is considered to be more adaptive and reflects on acute intrinsic and extrinsic stimuli. When excluding one or more measures of HR deficiency (e.g., LST, MS3 or RS3) in our approach, minor changes were observed in our classification. Interestingly, upon excluding genomic scars (HRD, LST, and AI) from the analysis, 33% of the tumours in Cluster s4 were reassigned to Cluster s2 (i.e., HR proficient) instead. This suggests that each cluster is driven by a unique set of features, as demonstrated in Figure 2B, and that genomic measures capturing HR deficiency, although correlated, capture subtly different, but important, types of genomic instability that could influence patient stratification.

Despite the differing breast cancer composition between ICGC (all breast cancer subtypes) and SCAN-B (only triple-negative), several clustering profiles were similar, particularly pertaining to HR deficiency and proficiency. The alignment of ICGC Cluster i5 and SCAN-B Cluster s4 was peculiar in that 95% of the cases in ICGC Cluster i5 were ER-positive. This indicates that in a small percentage of ER-positive breast cancers, the aetiology of HR deficiency resembles that of HR deficiency in TNBCs with *BRCA2*, *RAD51C* and *PALB2* alterations. None of the 20 ER-positive ICGC Cluster i5 cases had mutations in *ER*, although we did observe one case with an *NRAS* and another case with a *PIK3CA* mutation, both associated with resistance to endocrine therapy [41,42]. Taken together, these findings demonstrate how a robust integrative approach can lead to biologically relevant clusters with clear translational implications [20,43].

## 4. Materials and Methods

### 4.1. Permutation-Based Forest Clustering Algorithm

The permutation-based approach builds on the algorithm previously outlined for deriving a forest distance [31]. By independently sampling from a univariate empirical marginal distribution of the real data, a synthetic dataset composed of half synthetic and real data is generated. A forest classifier is then applied, dichotomising real and synthetic data, to derive a measure of similarity for the real data in the classification. The proximity matrix, defined as $S,s_{ij}\in {\left[0,1\right]}^{n\times n}$, gives the pairwise similarity distance between all N data points, both real and synthetic. After a forest is constructed, the proximity matrix, with values initialised at zero, can be calculated by passing the data down each tree in the forest. If two samples i and j fall in the same terminal node of a tree, their similarity $s_{ij}$ is increased by 1. Once finished, S is divided by the number of trees to normalise the distances between 0 and 1. The proximity matrix is turned into a dissimilarity matrix D, whereby $d_{ij}=\sqrt{1-s_{ij}}$. After omitting the synthetic data from D, the matrix can be used as input for traditional clustering algorithms. Our implementation of the permutation-based forest clustering is available at https://github.com/cancerbioinformatics/lumbRjacks (accessed on 25 February 2021).

Note that a proximity matrix of a forest is a function of the synthetic data generation and hence is subject to Monte Carlo variation. To produce stable estimates, dissimilarity matrices D and VIMs are averaged over many forests. Once the final forest distance has been derived, clustering in the full data space is performed using hierarchical consensus clustering with Ward linkage [44]. In all forest algorithms, the replacement scheme for sampling for each tree in the base classifiers was set to false to minimise category size bias, as outlined in [23]. The subsampling size was kept at the default level of 0.632 times the original dataset size.

Variable importance is assessed by the Gini impurity criterion index [22] and informs on the predictive power of a feature. To measure the unconditional permutation importance, data is passed down each tree in the forest and the accuracy of predicting between the real and synthetic data is recorded. A permutation is performed to break the association between the feature and the label. By averaging the difference in accuracy before and after the permutation, an unconditional VIM is calculated. To assess variable importance in complex data sets with correlated features, a conditional permutation importance framework is employed. The values of a feature are conditionally permuted on the groupings of every other feature, one at a time, thereby breaking the dependent structure of the correlated features. Be measuring the partial correlation of one feature while controlling for the effect of another, a true variable importance measure can be obtained. In concordance with Diaz-Uriarte and Alvarez de Andres [45], VIMs were not scaled.

### 4.2. Evaluation Metrics

To assess the agreement between clustering approaches, the Adjusted Rand Index (ARI) [46], Cluster purity [47], and Baker’s Gamma [48] were calculated. ARI measures the agreement between two classifications, even when the number of classes is different. Cluster purity measures the extent of which a cluster is comprised of a single class and Baker’s Gamma is used to compare the similarity between two dendrograms. Internal consistencies were evaluated by calculating the Calinski–Harabasz Index (CHI) and feature purity, which represent the degree of homogeneity within the clusters given either numerical or categorical features, respectively. During simulation studies, out-of-the-bag (OOB) errors are reported as a measure of prediction error. Each evaluation metric is described in detail in the Supplementary Methods.

### 4.3. Datasets

Two simulation studies were performed to assess potential biases in classification performance as a result of cardinality and correlation. A detailed description of how the data for these studies was generated is provided in the Supplementary Methods.

For the performance evaluation of the permutation-based framework for RF, RRF and CIF using real-world data, nine benchmarking classification datasets from the UCI Machine Learning Repository were tested [34]. Datasets were chosen to include varying numbers of samples, features and classes. Three were composed exclusively of numerical features, including Iris (“Iris”), Wisconsin Breast Cancer (“Breast”) and Glass Identification (“Glass”). Another three consisted of categorical features of varying cardinality: Soybean (“Soybean”), Zoo (“Zoo”) and Congressional Voting Records (“Voting”). Three more mixed-type datasets were included, namely Statlog German Credit (“Credit”), Indian Liver Patient (“Liver”) and Census Income (“Census”). Analyses were restricted to complete cases and true labels were withheld from the forest clustering to augment an unsupervised learning task.

A forest clustering algorithm was used to classify breast cancers from two multitype data breast cancer cohorts, namely (i) the 560 breast cancer study [3], consisting of 560 cancers and 147 available features (referred to as ICGC), and (ii) 241 TNBCs with 50 mixed-type features from the Sweden Cancerome Analysis Network—Breast (SCAN-B) initiative [8] (Table S3). A description of the parameters used for each of these datasets can be found in the Supplementary Methods.

## 5. Conclusions

Altogether, this work demonstrates the applicability of a permutation framework for forest methods in identifying structures in high-dimensional mixed-type data. The framework is flexible, scalable, and applicable to a large variety of studies. Our analyses indicate the complexity of breast cancer aetiologies and the importance of high-dimensional mixed-data type classification in identifying clinically relevant clusters. We expect that our approach will continue to unravel the intertumour heterogeneity and ultimately contribute to patient selection in the clinical trial setting.

## Data Availability

The data presented in this study are openly available at doi:10.1038/s41591-019-0582-4, doi:10.1200/PO.17.00135 and doi:10.1038/nature17676.

## Supplementary Materials

The following are available online at https://www.mdpi.com/2072-6694/13/5/991/s1, Figure S1: Diagram illustrating the permutation-based random forest classification methodology, Table S1: Benchmarking of permutation-based random forest classification methods in data obtained from the UCI Machine Learning Repository, Table S2: Rearrangement signature rank in ICGC data for each forest classification approach, Table S3: List of available features for the ICGC and SCAN-B cohorts, Table S4: Clinicopathological characteristics in RF clusters in ICGC, Table S5: Clinicopathological characteristics in RRF clusters in ICGC, Table S6: Clinicopathological characteristics in CIF clusters in ICGC and SCAN-B. Supplementary Methods: Describing the various evaluation metrics employed, the simulation studies, and how the permutation-based forest clustering methods were applied to each breast cancer dataset.
