# Supplementary material for: Random Forest Modelling of High-Dimensional Mixed-Type Data for Breast Cancer Classification
Source: Cancers (Basel). 2021 Feb 27;13(5):991. doi: 10.3390/cancers13050991 (PMC7956671; doi:10.3390/cancers13050991)
Supplement: Supplementary file 1 [file cancers-13-00991-s001.zip › Supplementary Methods.docx]

Supplementary Methods

Evaluation Metrics

To assess the agreement between clustering approaches, three external metrics were used, namely the Adjusted Rand Index (ARI) [1], Cluster purity, and Baker’s Gamma [2].

The ARI measures level of agreement between two partitions, even when the partitions being compared have different numbers of cluster labels. More specifically, given two partitions of the data $A$ and $B$ the ARI is given as:

$$ARI\left( A,B \right)=\frac{\sum_{i}^{K_{A}} \sum_{j}^{K_{B}} \binom{n_{ij}}{2}-\left( \sum_{i}^{K_{A}} \binom{n_{i}}{2}\sum_{j}^{K_{B}} \binom{n_{j}}{2} \right)/\binom{N}{2}}{\frac{1}{2}\left( \sum_{i}^{K_{A}} \binom{n_{i}}{2}+\sum_{j}^{K_{B}} \binom{n_{j}}{2} \right)-\left( \sum_{i}^{K_{A}} \binom{n_{i}}{2}\sum_{j}^{K_{B}} \binom{n_{j}}{2} \right)/\binom{N}{2}}$$

where $K_{A}$ and $K_{B}$ are the number of clusters in $A$ and $B$ respectively, $n_{i}$ and $n_{j}$ are the numbers of data points of individuals in clusters $i$ and $j$ of partitions $A$ and $B$, respectively, $n_{ij}$ is the number of data points in both cluster $i$ of partition $A$ and cluster $j$ of partition $B$, and $N$ is the total number of data points. The range of values for ARI is $[0,1]$, with higher values indicating more agreement in the cluster labels between partitions $A$ and $B$.

Cluster purity was measured to capture to which extent clusters contain one class. The range for cluster purity is [0,1], and is calculated as follows:

$$ClusterPurity=\frac{\sum_{k=1}^{K} a_{k}}{N}$$

where $N$ is the total number of data points, and $a_{k}$ is the number of data points with the majority class label of cluster $k$ [3].

To compare the structure (similarity) of two dendrograms built on the same dataset, the Baker’s Gamma [2] is calculated, which is defined as the rank correlation between the stages at which pairs of objects combine in each of the two trees.

To evaluate the internal consistency of clusters the Calinski-Harabasz Index (CHI) and feature purity were calculated.

CHI measures the quality of the clustering of numeric features to produce individual clusters that are tightly packed and well separated from each other, and is calculated as:

$$CHI\left( A \right)=\frac{SS_{B}}{SS_{W}}\times\frac{N-K}{K-1}$$

where $SS_{W}$ gives the within cluster sum-of-squares:

$$SS_{W}=\sum_{i}^{K} \sum_{x\in K_{i}} \left| \left| x-m_{i} \right| \right|^{2}$$

And $SS_{B}$ gives the between cluster sum-of-squares:

$$SS_{B}=\sum_{i}^{K} n_{i}\left| \left| m_{i}-C \right| \right|$$

Here, $K$ is the number of clusters, $K_{i}$ is the ith cluster, $m_{i}$ is the centroid of the $i^{th}$ cluster and $C$ is the global centroid of the data. The term on the right of CHI ensures that it scales correctly across different values of $K$; $SS_{B}$ has $K-1$ degrees of freedom, while $SS_{W}$ has $N-K$. Thus, the ratio of SSB over SSW should be the biggest at the optimal clustering size.

For the feature purity, the frequency that categorical features for a particular cluster group are homogenous above a threshold $p$, is defined as:

$$FeaturePurity=\sum_{k}^{K} \sum_{c}^{C} \sum_{f}^{F} \frac{I_{\hat{p}>p}}{n_{k}}$$

Where

$$\hat{p_{f,k,c}}=\frac{n_{f,k,c}}{n_{k}}$$

Here, $n_{f,k,c}$ is the number of data points assigned to class $c$, in cluster $k$, for feature $f$, and $n_{k}$ is the number of data points in cluster $k$. $\hat{p_{f,k,c}}$ gives the proportion of a feature that is completely assigned to a single class $c$ for cluster $k$. $I_{\hat{p}>p}$ is the indicator function, giving 1 if $\hat{p}$ is greater than the threshold $p$. When summing across all features $f$, clusters $k$ and classes $c$, $FeaturePurity$ gives the average feature purity for an entire dataset.

Simulation studies

Two simulation studies were conducted. First, a two-class classification dataset was generated, and the true labels set aside to later be compared to the cluster labels. The data consisted of nine categorical features $X_{1,\ldots,9}$ with ranging cardinality $card=\left( 2,\ldots,10 \right)$. Each feature was given a certain level of signal, $sig=\left( 0.9,\ldots,0.1 \right)$, which was inversely proportional to the cardinality of that feature and reflects the influence of a feature on the true label. For example, feature $X_{1}$ with cardinality 2 contained the most signal (0.9) relating to the true label, while feature $X_{9}$ had virtually no signal (0.1) and was effectively noise. On each iteration of the simulation, two classes were generated with 50 data points each, and the entire simulation was run in 100 iterations.

To generate features, a vector of uniform probabilities $u_{card.N}=\frac{1}{N}\left( \overset{\text{N}}{\overbrace{1,\ldots,1}} \right)$ was created for selecting a category for each feature, i.e. if the cardinality of a feature is 4 then the initial probability of selecting the 4 categories in the feature was 0.25. Next, Gaussian noise $\epsilon\sim N\left( 0,2\times sig\left[ N \right]^{2} \right)$ was added to each initial, centred at zero and with variance proportional to the signal of that feature giving $p_{card}=u_{card.N}+$ ε. For features with small cardinality and high signal, this created one category in each feature that was more likely to be chosen than any others, and vice versa for features with high cardinality/low signal where all categories were almost equally likely to be sampled. After applying a standard softmax function, this vector was used as a probability distribution for selecting categories of that feature.

To examine the effect of correlated features on the VIMs evaluation metrics, correlation simulation studies were performed. Data were generated from a linear model of twelve features $y=\beta_{1}\dot{x_{i,1}}+\ldots+\beta_{12}\dot{x_{i,12}}$ [4]. These data were sampled from a multivariate Gaussian distribution $X_{1},\ldots,X_{12}\sim N\left( 0,\Sigma\right)$. In the covariance matrix $\Sigma$, $X_{1},\ldots,X_{4}$ are block-correlated with $\sigma_{i,i’}=0.9$ for $i \neq i’ \leq4$, while $X_{5},\ldots,X_{12}$ are generated independently with $\sigma_{i,i’}=0$ for $i,i’\geq4$. As all the features have unit variance $\sigma_{i,i}=1$, the covariance equals the correlation.

To control the influence of each feature, the $\beta_{i}$ regression coefficients were given values $\beta=\left( 5, 5, 2, 0,-5,-5,-2, 0, 0, 0, 0, 0 \right)$. Here $\beta_{1},\ldots,\beta_{4}$ and $\beta_{5},\ldots,\beta_{8}$ share the same coefficient pattern, but only $\beta_{1},\ldots,\beta_{4}$ are correlated. $\beta_{9},\ldots,\beta_{12}$ were given zero coefficient values and were treated as noise in the generated data $X$. A binary label was modelled by feeding the data $X$ through the logistic function and a threshold applied at 0.5 to determine the true binary labels:

$$class labels=\left\{ \begin{aligned} \frac{e^{X\beta}}{1+e^{X\beta}}>0.5,1 \\ else, 0 \end{aligned} \right.$$

Both simulation studies were used to test RF, RFF, uncondCIF and condCIF. Parameters included $no.forests=100$, $no.trees=200$ across 100 simulations. For each simulation, the number of clusters was presumed to be two. The forest distance was fed directly into hierarchical clustering with Ward linkage. The resulting dendrogram was empirically cut and evaluated. Additionally, on each run the VIM and out-of-the-bag (OOB) errors were measured. In the conditional permutation, VIMs are superior to the unconditional measure in identifying the true influential predictors $X_{1},X_{2},X_{5},X_{6}$ in the classification setting [4]. The results were a function of the parameter mtry, as the incorporation of more features in each tree allowed the conditional permutation variable importance measures to more precisely deconvolute the feature interactions. To evaluate whether these findings translate to the unsupervised setting, mtry of 1, 3, and 8 were used in both simulation studies.

Evaluation of forest clustering methods in breast cancer datasets

Evaluation of our methods on real data was done using two complex, multi-type data breast cancer cohorts, namely (i) the 560 breast cancer study [5], consisting of 560 cancers and 147 available features (referred to as ICGC), and (ii) 241 TNBCs with 50 mixed-type features from the Sweden Cancerome Analysis Network - Breast (SCAN-B) initiative [6]. The performance of the permutation-based forest algorithms was examined using six rearrangement signatures, each with a proposed different aetiology [5].

To establish whether similar clusters were found, different forest clustering methods were applied to the six published rearrangement signatures. Using a total of 533 out of the 560 cases, RF, RFF, uncondCIF and condCIF were applied to the data, with the settings $mtry=3$; $no.tree=2000$; $no.forests=100$. Identical to the original methodology, consensus clustering of the forest distances was done using number of repetitions = 1000; pItem = 0.9 (resampling frequency data points); Euclidean distance metric; and Ward linkage method. In parallel, the hierarchical clustering from [5] (“original clustering”) was repeated on the 533 cases to allow comparisons of the dendrograms, using number of repetitions = 1000; pItem = 0.9; pFeature = 0.9 (feature resampling frequency); Pearson distance metric; and Ward linkage method.

Next, additional clinico-pathological and molecular features were incorporated by applying the permutation-based forest approach to 147 features in ICGC, and 50 in SCAN-B using mtry=14; ntree=2000; nforests=100. For consensus clustering, the following parameters were used: number of repetitions = 1000; pItem = 0.9; Euclidean distance metric; Ward linkage.

References

1. Santos, J.M.; Embrechts, M. On the Use of the Adjusted Rand Index as a Metric for Evaluating Supervised Classification. In Proceedings of Proceedings of the 19th International Conference on Artificial Neural Networks: Part II, Limassol, Cyprus; pp. 175–184.

2. Baker, F.B. Stability of two hierarchical grouping techniques Case I: Sensitivity to data errors. . Journal of the American Statistical Association, 1974, 69, 440-445.

3. Manning, C.D.; Raghavan, P.; Schuetze, H. Introduction to Information Retrieval; New York, NY, USA 2008.

4. Strobl, C.; Boulesteix, A.-L.; Kneib, T.; Augustin, T.; Zeileis, A. Conditional variable importance for random forests. BMC Bioinformatics 2008, 9, 307, doi:10.1186/1471-2105-9-307.

5. Nik-Zainal, S.; Davies, H.; Staaf, J.; Ramakrishna, M.; Glodzik, D.; Zou, X.; Martincorena, I.; Alexandrov, L.B.; Martin, S.; Wedge, D.C., et al. Landscape of somatic mutations in 560 breast cancer whole-genome sequences. Nature 2016, 534, 47-54, doi:10.1038/nature17676.

6. Staaf, J.; Glodzik, D.; Bosch, A.; Vallon-Christersson, J.; Reutersward, C.; Hakkinen, J.; Degasperi, A.; Amarante, T.D.; Saal, L.H.; Hegardt, C., et al. Whole-genome sequencing of triple-negative breast cancers in a population-based clinical study. Nat Med 2019, 25, 1526-1533, doi:10.1038/s41591-019-0582-4.
